# Production of Sulphurets on Metallic Bodies Struck by Lightning

**Published:** 1846-11

**Authors:** 


*Production of Sulphurets on Metallic bodies struck by lightning.*—Nothing is more common than to hear, when persons or buildings have been struck by lightning, that there was a strong odour of sulphur in and around the spot. This odour has, however, been generally referred to a partial combustion of organic matter. How an imponderable like the electric fluid can contain, be combined with, or elicit sulphur either from the atmosphere or by some unknown method of synthesis, it is not easy to understand ; but M. Bonjean appears to be the first chemist who has proved that sulphur is actually evolved by the effect of a stroke of lightning.

On Sunday, June 14, 1846, a church near Chumbery was struck by lightning. The interior was filled with a thick smoke, and the smell was compared to that of gunpowder. M. Bonjean having been informed that certain gilt articles had been blackened by the electric fluid, examined the place the following day. He found that the gilt frame of a large picture was almost entirely blackened in its longitudinal and transverse portions. Six gilt chandeliers were blackened, exactly like copper immersed in sulphuretted hydrogen gas; while a cross, guilt exactly like the chandeliers, and placed in the midst of them, had not undergone any change. A portion of the dark film was scratched from one of the tarnished chandeliers, and digested in pure nitro-muriatic acid. The addition of a salt of barytes to the diluted solution indicated clearly the presence of sulphuric acid. It would thus appear that the electric fluid is accompanied with sulphur in some state of combination, probably as sulphuretted hydrogen. If it were sulphurous acid, oxidable metals would be transformed to sulphites and sulphates. The odour, therefore, so frequently observed, is probably due to the presence of the above mentioned gas.

It may be objected that gold is not chemically attacked by sulphureous vapours; but M. Bonjean contends that so far back as 1838, he proved that gold when exposed to the vapour of sulphureous waters, is liable to become tarnished and transformed in the course of fifteen or twenty days, to a sulphuret, like silver, lead, copper, &c. The only difference is, that these metals, being much more oxidable, the reaction caused by sulphur becomes more speedily evident—*Comptes Rendus.*

[M. Bonjean's observation on this chemical effect of an electric stroke, is novel and interesting. We take leave to doubt, however, whether sulphur vapours, under any circumstances, will tarnish *pure* gold. Much of the gold sold for gilding is alloyed with copper, and we apprehend that we have, in the extensive use of this alloy, or in the use of Dutch lead itself, a full explanation of this alleged action of sulphur on gold, in this and other instances. We have known *pure* gold to preserve its polish and brightness in a London atmosphere, where sulphur abounds, for seventeen years. The gold plates used by the Egyptians for covering the features of their mummies, and their mummy cases, is, if we except superficial dirt, as bright as when first laid down, 3,000 years ago.]—*London Med. Gaz.*

